# Correction to ‘BMAL1 deficiency promotes skeletal mandibular hypoplasia via OPG downregulation’

**DOI:** 10.1111/cpr.13750

**Published:** 2024-09-20

Zhou X, Yu R, Long Y, et al. BMAL1 deficiency promotes skeletal mandibular hypoplasia via OPG downregulation. *Cell Prolif*. 2018;51:e12470. doi:10.1111/cpr.12470.

The Western blot bands of OPG in Figure 4A of BMAL1‐overexpressed BMSCs and BMAL1 in Figure 4B of BMAL1‐overexpressed MC3T3‐E1 cell line were incorrectly copied.

Corrected Figure 4 is provided below. The correction does not alter any findings and conclusions reported in this article.

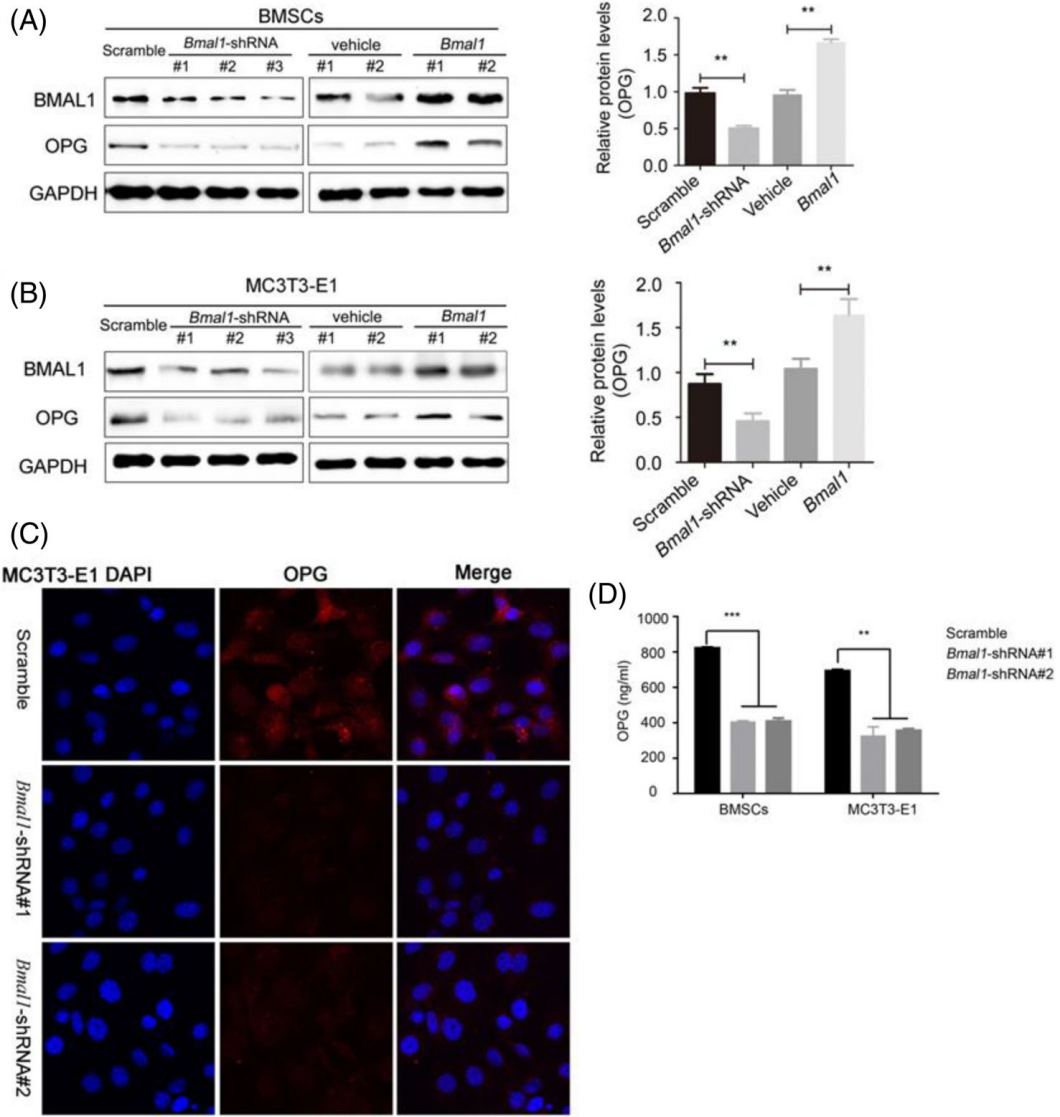

We apologize for this error.

